# Ginsenoside Rg1 Attenuates Muscle Atrophy in Hyperglycemic Conditions, Inactivity and Protein Deprivation Models via AKT/mTOR Pathway Activation

**DOI:** 10.2174/0115665240355315250414051525

**Published:** 2025-04-23

**Authors:** Xu He, Yan Li, Jun Chen, Li Zhang, Yan Huang, Ying Zhou, Jing Li

**Affiliations:** 1 Medical Faculty, Kunming University of Science and Technology, Kunming, Yunnan, China;; 2 Department of Geriatrics, The First People’s Hospital of Yunnan Province, Kunming, Yunnan, China;; 3 The Affiliated Hospital of Kunming University of Science and Technology, Kunming, Yunnan, China

**Keywords:** Ginsenoside Rg1, Muscle Atrophy, Hyperglycemia, AKT/mTOR Pathway, C2C12 Myoblasts, Inactivity, Protein Restriction

## Abstract

**Background:**

Muscle atrophy, a debilitating condition prevalent in diabetes and extended periods of immobilization, lacks robust therapeutic strategies. This investigation examines ginsenoside Rg1's therapeutic potential in counteracting muscle atrophy under hyperglycemic conditions and in experimental models of immobilization and dietary protein restriction.

**Methods:**

C2C12 murine myoblasts were cultured under variable glucose concentrations and treated with or without Rg1. Multiple cellular parameters were evaluated, including cell viability, apoptotic indices, cell cycle distribution, and protein synthesis rates. The activation status of the protein kinase B (AKT)/mammalian target of rapamycin (mTOR) signaling cascade and expression of atrophy-related markers were quantified using qRT-PCR and Western blot analyses. In parallel animal studies, rats were subjected to either immobilization or protein restriction protocols, with or without Rg1 administration. Muscle function, mass, and relevant biomarkers were evaluated.

**Results and Discussion:**

Hyperglycemic conditions significantly compromised C2C12 myoblast viability, triggered apoptotic pathways, and disrupted normal cell cycle progression. Rg1 administration effectively attenuated these detrimental effects through enhanced AKT/mTOR pathway activation, upregulation of Myogenin (MyoG) expression, and suppression of atrophy-associated markers. In the rat models, Rg1 supplementation significantly ameliorated muscle deterioration, maintaining muscle mass, contractile force, and exercise tolerance, while simultaneously modulating atrophy signaling pathways and attenuating inflammatory responses. The protective effects of Rg1 were abrogated after the co-treatment with an AKT inhibitor.

**Conclusion:**

Ginsenoside Rg1 exhibits significant protective properties against muscle atrophy under hyperglycemic conditions and in experimental models of immobilization and protein restriction, primarily mediated through activation of the AKT/mTOR signaling pathway. These findings establish Rg1 as a promising therapeutic candidate for the treatment of muscle atrophy.

## 1 INTRODUCTION

The progressive deterioration of muscle mass and function represents a major public health challenge, particularly in an era of aging demographics and increasingly sedentary lifestyles [1]. Multiple factors can precipitate this condition, including physiological aging, physical inactivity, nutritional deficiencies, and various pathological conditions [2]. The hallmarks of muscle atrophy include reduced muscle fiber dimensions, diminished protein content, and impaired force generation capacity [3]. Risk factors encompass prolonged immobilization, insufficient protein consumption, and chronic inflammatory conditions [4]. While current therapeutic strategies predominantly focus on resistance training and nutritional supplementation [5], these interventions have limited efficacy, especially in patients who are physically incapacitated or experience compromised appetite [6].

Multiple factors, including physical activity, protein intake, chronic inflammation, and hyperglycemia, exert profound effects on muscle atrophy through distinct molecular mechanisms. Regular exercise, especially resistance training, enhances muscle protein synthesis and satellite cell activation, thereby facilitating muscle regeneration and hypertrophy [7,8]. Optimal protein consumption ensures the availability of essential amino acids for muscle protein synthesis, with leucine serving as a primary activator of the mTOR pathway to orchestrate muscle growth [9,10]. In contrast, chronic inflammatory states, commonly observed in obesity and autoimmune disorders, compromise muscle regeneration through elevated pro-inflammatory cytokines, particularly TNF-α and IL-6. These cytokines subsequently trigger protein degradation cascades, notably the ubiquitin-proteasome system [11,12]. Hyperglycemic conditions in diabetic patients amplify muscle atrophy by generating oxidative stress and advanced glycation end-products (AGEs). These factors disrupt insulin signaling and attenuate PI3K/Akt pathway activation, ultimately compromising protein synthesis [13-15]. This relationship is exemplified in experimental studies with diabetic rats, where sustained hyperglycemia resulted in a significant 25% reduction in muscle fiber cross-sectional area and a 40% decrease in myofibrillar protein content compared to control subjects, demonstrating the substantial impact of elevated blood glucose on muscle integrity [16].

Ginsenoside Rg1, a principal bioactive constituent of *Panax ginseng*, has garnered significant attention in the scientific community for anti-aging research due to its diverse pharmacological effects and therapeutic potential [17]. This triterpenoid saponin exhibits remarkable influences across multiple physiological systems, particularly in neuroprotection, cardiovascular function, and muscle metabolism [18]. In muscle physiology, Rg1 demonstrates considerable promise in attenuating muscle atrophy and enhancing regenerative processes. A previous study has shown that ginsenoside Rg1 administration enhances skeletal muscle oxidative metabolism and promotes an anabolic response *in vivo* [19], suggesting its potential role in muscle function and metabolism. Furthermore, Rg1 activates the PI3K/Akt/mTOR signaling cascade, a fundamental regulator of protein synthesis and muscle hypertrophy [20]. Regarding metabolic regulation, Rg1 exhibits significant insulin-sensitizing properties, enhancing muscular glucose uptake. Research in diabetic mouse models has demonstrated that Rg1 treatment induces a 30% increase in glucose transporter 4 (GLUT4) membrane translocation in skeletal muscle, significantly improving glucose utilization [21]. Additionally, the well-documented anti-inflammatory properties of Rg1 [22,23] may contribute to muscle preservation by suppressing chronic low-grade inflammation, a common feature in muscle wasting disorders. These multifaceted effects of Rg1 on muscle homeostasis and metabolic regulation highlight its potential as a therapeutic agent for muscle disorders and metabolic dysfunction.

This study investigates the potential of ginsenoside Rg1 in mitigating muscle atrophy under conditions of hyperglycemia, inactivity, and protein restriction. C2C12 mouse myoblasts were utilized to assess the effects of Rg1 on cell viability, apoptosis, and protein synthesis in high-glucose environments. Rat models of inactivity and protein restriction were employed to evaluate Rg1's influence on muscle function and mass. The research focuses on the AKT/mTOR signaling pathway and atrophy markers to elucidate the protective mechanisms of Rg1. The findings from this study could have significant implications for the development of novel interventions aimed at preventing or alleviating muscle atrophy in various clinical contexts, including diabetes management, prolonged bed rest, and malnutrition-related conditions.

## 2 MATERIALS AND METHODS

### 2.1 Cell Culture and Treatments

C2C12 mouse myoblasts (PromoCell, Heidelberg, Germany) were cultured in DMEM supplemented with 10% FBS and 1% penicillin/streptomycin at 37°C in a 5% CO_2_ incubator (Thermo Scientific Heracell 150i). For hyperglycemia experiments, cells were exposed to varying glucose concentrations (5.5 mM, 22.5 mM, and 50 mM) for 48 hours, with mannitol added to the lower glucose conditions to equalize osmotic pressure. In experiments involving ginsenoside Rg1, cells were treated with various concentrations of Rg1 (0.1 μM to 100 μM) under both normal (5.5 mM) and high (50 mM) glucose conditions for 48 hours. Ginsenoside Rg1 (purity ≥98%) was sourced from Sigma-Aldrich (St. Louis, MO, USA). For AKT inhibition experiments, 10 μmol/L GSK690693 (MedChemExpress, Monmouth Junction, NJ, USA) was utilized.

### 2.2 Cell Growth Assay

Cell growth was evaluated using the XTT Cell Proliferation Assay Kit (Abcam, Cambridge, UK). C2C12 cells were seeded in 96-well plates at a density of 2 × 10^3 cells per well. Following the treatments, cell growth was measured at 24, 48, and 72 hours in accordance with the manufacturer's instructions. Absorbance was recorded at 450 nm using a Synergy H1 microplate reader (BioTek Instruments, Winooski, VT, USA).

### 2.3 Flow Cytometry Analysis

Apoptosis was examined using the FITC Annexin V Apoptosis Detection Kit with PI (BioLegend, San Diego, CA, USA), following the supplier's instructions. For cell cycle analysis, cells were fixed in 70% ethanol overnight at 4°C and subsequently stained with PI/RNase staining solution (Zeye Biotech, Shanghai, China). Analyses were conducted using a CytoFLEX flow cytometer (Beckman Coulter, Brea, CA, USA), recording a minimum of 10,000 events per sample. Data analysis was performed using CytExpert software.

### 2.4 Quantitative Real-Time PCR (qRT-PCR)

Total RNA was extracted from tissue or cell samples using the RNeasy Mini Kit (Qiagen, Hilden, Germany). The RNA was then reverse transcribed using the High-Capacity cDNA Reverse Transcription Kit (Applied Biosystems, Foster City, CA, USA). Quantitative real-time PCR was performed with PowerUp SYBR Green Master Mix (Applied Biosystems) on a QuantStudio 3 Real-Time PCR System (Thermo Fisher Scientific). Relative gene expression was calculated using the 2^-ΔΔCt method, employing beta-actin as the internal control.

### 2.5 Western Blot Analysis

Cells or tissue samples were lysed using RIPA buffer that contained protease and phosphatase inhibitors (Zeye Biotech, Shanghai, China). Protein concentrations were determined with the Pierce BCA Protein Assay Kit (Thermo Fisher Scientific). Approximately 20 μg of protein samples were separated by SDS-PAGE and subsequently transferred to PVDF membranes. The following primary antibodies were utilized for target detection: anti-AKT (1:1000, Cell Signaling Technology), anti-phospho-AKT (Ser473) (1:1000, Cell Signaling Technology), anti-mTOR (1:1000, Abcam), anti-phospho-mTOR (Ser2448) (1:1000, Abcam), anti-Myogenin (MyoG, a muscle-specific transcription factor; 1:500, Santa Cruz Biotechnology), anti-Muscle Atrophy F-box (MAFbx/Atrogin-1; 1:1000, Proteintech), anti-Muscle RING Finger-1 (MuRF1; 1:1000, Proteintech), and anti-Glyceraldehyde 3-phosphate dehydrogenase (GAPDH; 1:5000, Sigma-Aldrich). HRP-conjugated secondary antibodies (Jackson ImmunoResearch) were applied at a dilution of 1:5000. Protein bands were visualized using SuperSignal West Pico PLUS Chemiluminescent Substrate (Thermo Fisher Scientific) and quantified with Image Lab software (Bio-Rad).

### 2.6 *De Novo* Protein Synthesis Assay

Quantification of protein synthesis was performed using the OPP Protein Synthesis Assay Kit (Cayman Chemical, Ann Arbor, MI, USA). C2C12 myoblasts were cultured in 96-well plates under the specified experimental conditions. Following treatment, cells underwent sequential processes of labeling reagent incubation, fixation, and permeabilization in accordance with the manufacturer's protocol. Image acquisition was conducted using an EVOS M7000 Imaging System (Thermo Fisher Scientific) equipped with corresponding filter configurations. Quantitative analysis of fluorescence intensity was performed using Cellomics software (Thermo Fisher Scientific), enabling automated high-content image analysis of protein synthesis levels.

### 2.7 Animal Studies

Male Sprague-Dawley rats (6 months old, weighing 180-220g) were acquired from Kunming Medical University (Kunming, Yunnan, China) and maintained in a climate-controlled environment (22 ± 2°C) with a standardized 12-hour light/dark cycle and unrestricted access to water. All experimental procedures received approval from the Ethical Review Committee of The First People's Hospital of Yunnan Province (NO.2020KYS074). The study comprised seven experimental groups (n=6 per group): Control, Inactive, Inactive+Rg1, Inactive+Rg1+GSK690693, Protein-restricted, Protein-restricted+Rg1, and Protein-restricted+Rg1+GSK690693. The inactivity model was implemented using specialized cages that prevented hindlimb weight-bearing while enabling unrestricted forelimb movement over a 30-day period. Dietary intervention consisted of either a low-protein diet (0.5% protein, 75.5% carbohydrate, 7.5% fat) for protein-restricted groups or a standard diet (18% protein, 58% carbohydrate, 7.5% fat; Research Diets, Inc., New Brunswick, NJ, USA) for other groups. Rg1 treatment groups received daily oral administration of ginsenoside Rg1 (50 mg/kg/day; MedChemExpress, Shanghai, China). GSK690693 groups received intramuscular injections of the AKT inhibitor (5 mg/kg; MedChemExpress) every three days. Following the 30-day experimental period, muscle performance assessments were conducted. Subsequently, blood and muscle tissue samples were collected after euthanasia by cervical dislocation.

### 2.8 Physical Performance Measurements

Gastrocnemius muscle diameter was measured using digital calipers (Mitutoyo, Kawasaki, Japan). Hindlimb grip strength was quantified using a specialized grip strength meter (IITC Life Science, Woodland Hills, CA, USA). The assessment protocol involved gentle backward tail traction while the animals gripped a force transducer-connected T-bar. Maximum force generation was recorded across five trials, with mandatory 1-minute rest intervals between attempts. The mean of the three highest measurements was utilized for analysis. Exercise endurance evaluation was conducted using an Exer-3/6 Open Treadmill apparatus (Columbus Instruments, Columbus, OH, USA). Prior to testing, the rats underwent a three-day acclimatization period on the treadmill. The testing protocol commenced at a speed of 10 m/min with a 5% incline, followed by progressive increments of 1 m/min every 2 minutes. Exhaustion criteria were defined as the inability to maintain running performance for 10 seconds despite mild electrical stimulation, at which point both time to exhaustion and total distance covered were documented.

### 2.9 Blood Biochemistry

Blood samples were collected via cardiac puncture under isoflurane anesthesia (Piramal Critical Care, Bethlehem, PA, USA). Serum was separated by centrifugation and analyzed for liver enzymes (ALT, AST), creatine kinase (CK), albumin, creatinine, and testosterone using a Cobas c501 automated biochemical analyzer (Roche Diagnostics, Basel, Switzerland).

### 2.10 Histological Analysis

Gastrocnemius muscles from hindlimb were fixed, embedded in paraffin, sectioned, and stained with a Hematoxylin & Eosin staining kit (Beyotime, Beijing, China). Images were captured using a BX53 light microscope (Olympus, Tokyo, Japan) equipped with a DP74 digital camera.

### 2.11 ELISA

Quantification of IL-6 levels in muscle tissue homogenates was performed using the Rat IL-6 Quantikine ELISA Kit (R&D Systems, Minneapolis, MN, USA). The assay protocol involved sequential addition of samples and standards to microplate wells pre-coated with capture antibody, followed by incubation with biotinylated detection antibody and streptavidin-HRP conjugate. Following the addition of substrate and stop solutions, absorbance measurements were obtained at 450 nm using a Synergy H1 microplate reader (BioTek Instruments). Final IL-6 concentrations were standardized to total protein content, which was determined using the Pierce BCA Protein Assay Kit (Thermo Fisher Scientific).

### 2.12 Statistical Analysis

All experimental data are expressed as mean ± standard deviation (SD). Statistical analyses were conducted using GraphPad Prism 9 software (GraphPad Software, San Diego, CA, USA). Comparisons among multiple groups were performed using one-way analysis of variance (ANOVA) followed by Tukey's post-hoc test. Interactions between two independent variables were evaluated using two-way ANOVA. Statistical significance was established at P < 0.05. (supplementary images)

## 3 RESULTS

### 3.1 Hyperglycemia Impairs C2C12 Myoblast Viability, Induces Apoptosis, and Alters Cell Cycle Progression

**Fig. (1):**
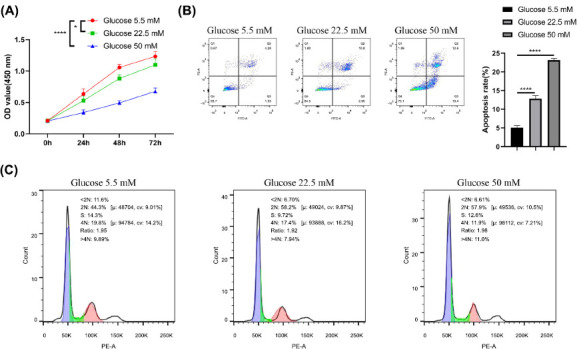
Hyperglycemia Impairs C2C12 Myoblast Viability, Induces Apoptosis, and Alters Cell Cycle Progression. C2C12 mouse myoblasts were exposed to varying glucose concentrations (5.5 mM, 22.5 mM, and 50 mM) for 48 hours, with mannitol added to lower glucose conditions to equalize osmotic pressure. (**A**) Cell viability assessed by CCK-8 assay over time. (**B**) Apoptotic rates determined by flow cytometry analysis. (**C**) Cell cycle progression analysis by flow cytometry, showing the proportion of cells in G1, S, and G2/M phases. Data represent mean ± SEM. N=3 independent experiments. *p<0.05, **p<0.01, ***p<0.001, ****p<0.0001 compared to 5.5 mM glucose condition.

To investigate hyperglycemia-induced muscle cell responses, C2C12 mouse myoblasts were exposed to normal (5.5 mM), moderate (22.5 mM), or high (50 mM) glucose concentrations for 48 hours. To control for osmotic effects, mannitol was added to the lower glucose conditions to maintain equivalent osmolarity across all treatments. The use of 50 mM glucose to model hyperglycemic conditions has been previously validated in studies examining glucose-induced cellular stress [24,25]. The 48-hour exposure period was selected to mimic chronic hyperglycemic conditions, allowing sufficient time for the development of cellular stress responses and metabolic adaptations that better represent the prolonged hyperglycemic state. Cell proliferation assessment via CCK-8 assay demonstrated a progressive decline in cell growth that correlated with both exposure time and glucose concentration (Fig. **1A**). Flow cytometric analysis revealed that increasing glucose concentrations induced a proportional elevation in apoptotic rates (Fig. **1B**). Cell cycle analysis identified significant alterations in phase distribution, characterized by glucose concentration-dependent accumulation of cells in G1 phase, accompanied by a concurrent reduction in S phase population (Fig. **1C**). These findings demonstrate that hyperglycemic conditions significantly compromise C2C12 myoblast function through multiple mechanisms, including reduced cellular viability, enhanced apoptotic response, and G1/S phase cell cycle arrest.

### 3.2 Hyperglycemia Modulates Protein Synthesis and Degradation Pathways in C2C12 Myoblasts

**Fig. (2):**
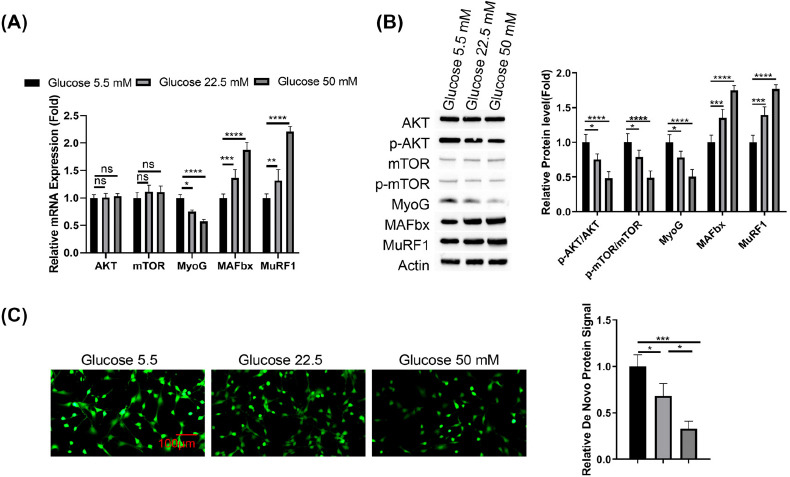
Hyperglycemia Modulates Protein Synthesis and Degradation Pathways in C2C12 Myoblasts. C2C12 myoblasts were exposed to varying glucose concentrations (5.5 mM, 22.5 mM, and 50 mM) for 48 hours. (**A**) qRT-PCR analysis of AKT, mTOR, MyoG, MAFbx, and MuRF1 mRNA expression. (**B**) Western blot analysis of phosphorylated and total AKT, mTOR, MyoG, MAFbx, and MuRF1 protein levels. (**C**) *De novo* protein synthesis assessed by Click-IT assay. Data represent mean ± SEM. N=3 independent experiments. *p<0.05, **p<0.01, ***p<0.001, ****p<0.0001 compared to 5.5 mM glucose condition.

To investigate the molecular mechanisms underlying high glucose-induced muscle atrophy, we examined gene expression and protein levels of key regulators in C2C12 myoblasts exposed to varying glucose concentrations. Quantitative RT-PCR analysis (Fig. **2A**) demonstrated that while AKT and mTOR mRNA levels remained stable across glucose concentrations, myogenin (MyoG, a muscle-specific transcription factor), exhibited a glucose-dependent decline. Concurrently, atrophy markers Muscle Atrophy F-box (MAFbx) and Muscle RING Finger-1 (MuRF1) showed progressive upregulation with increasing glucose levels. Western blot analysis (Fig. **2B**) revealed reduced phosphorylation of AKT and mTOR, decreased MyoG protein expression, and elevated MAFbx and MuRF1 protein levels under hyperglycemic conditions, despite unchanged total AKT and mTOR protein content. These findings indicate that hyperglycemia predominantly influences the activation state of AKT and mTOR signaling components. Assessment of *de novo* protein synthesis using Click-IT assay (Fig. **2C**) demonstrated a significant glucose concentration-dependent reduction in protein synthesis rates. These findings collectively indicate that hyperglycemia promotes muscle atrophy through dual mechanisms: suppression of protein synthesis pathways and enhancement of protein degradation processes in C2C12 myoblasts.

### 3.3 Concentration-Dependent Protective Effects of Ginsenoside Rg1 Against High Glucose-Induced Growth Inhibition and Apoptosis

**Fig. (3):**
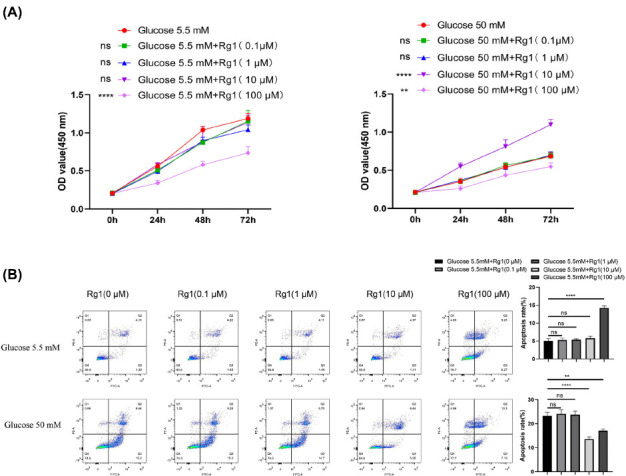
Ginsenoside Rg1 Exhibits Concentration-Specific Protection Against High Glucose-Induced Cell Proliferation Inhibition and Apoptosis. C2C12 myoblasts were treated with various concentrations of Rg1 (0.1 μM to 100 μM) under normal (5.5 mM) and high (50 mM) glucose conditions for 48 hours. (**A**) Cell proliferation assessed by CCK-8 assay. (**B**) Apoptotic rates determined by flow cytometry analysis. Data represent mean ± SEM. N=3 independent experiments. *p<0.05, **p<0.01, ***p<0.001, ****p<0.0001 compared to 0 μM Rg1 condition within each glucose group.

The protective potential of ginsenoside Rg1 against glucose-induced muscle atrophy was evaluated by treating C2C12 myoblasts with Rg1 concentrations ranging from 0.1 μM to 100 μM under both normal (5.5 mM) and high (50 mM) glucose conditions. Cell proliferation analysis via CCK-8 assay (Fig. **3A**) revealed distinct patterns under different glucose conditions. Under normal glucose conditions (5.5 mM), proliferation rates remained largely stable across most Rg1 concentrations, showing significant decline only at 100 μM. In high glucose conditions (50 mM), while overall proliferation was suppressed, treatment with 10 μM Rg1 demonstrated marked protective effects. However, this protection diminished at 100 μM Rg1, suggesting concentration-dependent toxicity. Apoptosis assessment through flow cytometry (Fig. **3B**) demonstrated that under normal glucose conditions, apoptotic rates remained minimal up to 10 μM Rg1 but increased significantly at 100 μM. In high glucose conditions, although baseline apoptotic rates were elevated, 10 μM Rg1 treatment significantly reduced apoptosis. This protective effect was not sustained at 100 μM Rg1. These results identify 10 μM as the optimal Rg1 concentration for protecting C2C12 myoblasts against hyperglycemia-induced cellular dysfunction, while higher concentrations proved ineffective or detrimental.

### 3.4 Rg1 Mitigates High Glucose-Induced Muscle Atrophy in C2C12 Myoblasts via AKT/mTOR Pathway Activation

**Fig. (4):**
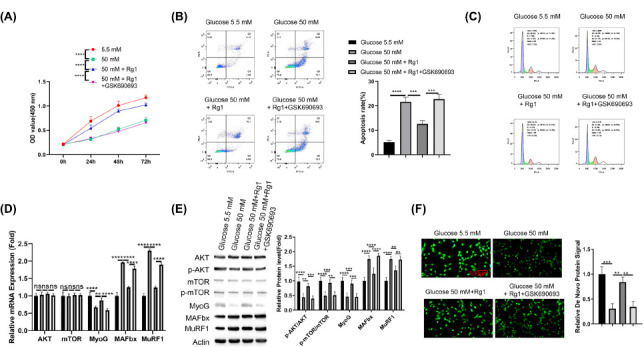
Rg1 Mitigates High Glucose-Induced Muscle Atrophy in C2C12 Myoblasts via AKT/mTOR Pathway Activation. C2C12 myoblasts were treated under various conditions: normal glucose (5.5 mM), high glucose (50 mM), high glucose with Rg1 (10 μM), and high glucose with Rg1 and AKT inhibitor GSK690693 (10 μmol/L) for 48 hours. (**A**) Cell proliferation assessed by CCK-8 assay. (**B**) Apoptotic rates determined by flow cytometry. (**C**) Cell cycle progression analysis. (**D**) qRT-PCR analysis of AKT, mTOR, MyoG, MAFbx, and MuRF1 mRNA expression. (**E**) Western blot analysis of phosphorylated and total AKT, mTOR, MyoG, MAFbx, and MuRF1 protein levels. (**F**) *De novo* protein synthesis assessed by Click-IT assay. Data represent mean ± SEM. N=3 independent experiments. *p<0.05, **p<0.01, ***p<0.001, ****p<0.0001 compared to normal glucose condition or as indicated.

To investigate the mechanism by which Rg1 protects against glucose-induced muscle atrophy, we examined C2C12 myoblasts under various conditions: normal glucose (5.5 mM), high glucose (50 mM), high glucose with Rg1 (10 μM), and high glucose with Rg1 and AKT inhibitor GSK690693 (10 μmol/L). CCK-8 assay (Fig. **4A**) showed that AKT inhibition abrogated the rescue effect of Rg1 under high glucose conditions. Flow cytometric analyses revealed that Rg1 significantly reduced hyperglycemia-induced apoptosis (Fig. **4B**) and restored normal cell cycle distribution (Fig. **4C**), with both effects nullified by AKT inhibition. Molecular analyses through qRT-PCR (Fig. **4D**) and Western blot (Fig. **4E**) showed that under hyperglycemic conditions, Rg1 treatment enhanced AKT and mTOR phosphorylation, elevated MyoG expression, and suppressed atrophy markers MAFbx and MuRF1. AKT inhibition prevented Rg1-mediated effects. Assessment of *de novo* protein synthesis (Fig. **4F**) further confirmed that AKT inhibition prevented Rg1's ability to restore protein synthesis under hyperglycemic conditions. These findings indicate that Rg1's protective effects against hyperglycemia-induced muscle atrophy are primarily mediated through AKT/mTOR pathway activation.

### 3.5 Rg1 Mitigates Muscle Deterioration and Physiological Changes Caused by Inactivity and Protein Restriction

**Fig. (5):**
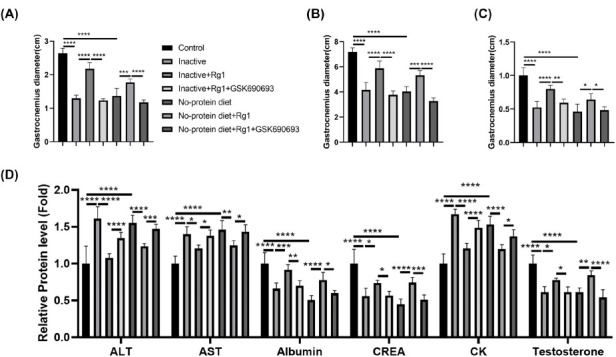
Rg1 Mitigates Muscle Deterioration and Physiological Changes Through AKT-Dependent Mechanisms. Rats were divided into seven groups: Control, Inactive, Inactive+Rg1, Inactive+Rg1+GSK690693, Protein-restricted, Protein-restricted+Rg1, and Protein-restricted+Rg1+GSK690693. (**A**) Gastrocnemius muscle diameter measurements. (**B**) Hindlimb grip strength assessment. (**C**) Relative exercise duration. (**D**) Blood biochemistry analysis including liver enzymes (ALT, AST), creatine kinase (CK), albumin, creatinine (CREA), and testosterone levels. Data represent mean ± SEM. N=6 animals per group. *p<0.05, **p<0.01, ***p<0.001, **p<0.0001.

To investigate the beneficial effects of Rg1 supplementation on muscle function under conditions of inactivity or protein restriction, and to elucidate the role of AKT signaling in these effects, we conducted comprehensive analyses across seven experimental groups: Control, Inactive, Inactive+Rg1, Inactive+ Rg1+GSK690693, Protein-restricted, Protein-restricted+Rg1, and Protein-restricted+Rg1+ GSK690693 (Fig. **5**). Gastrocnemius muscle diameter measurements showed significant reductions in both inactive and protein-restricted conditions compared to controls. Rg1 supplementation partially reversed this atrophy, and this protective effect was abolished by GSK690693 administration (Fig. **5A**). Similar results were observed in hindlimb grip strength assessments, where Rg1's capacity to preserve muscle strength was negated by AKT inhibition (Fig. **5B**). Exercise endurance, measured as relative exercise duration, decreased across all experimental conditions compared to controls. Rg1 supplementation improved exercise capacity in both inactive and protein-restricted groups, while GSK690693 co-administration prevented these beneficial effects (Fig. **5C**).

Blood biochemistry analysis revealed significant alterations across multiple markers (Fig. **5D**). Liver function markers alanine aminotransferase (ALT) and aspartate aminotransferase (AST) showed elevated levels in both inactive and protein-restricted groups compared to controls, indicating hepatic stress. Creatine kinase (CK), a marker of muscle damage, demonstrated similar elevation patterns. Rg1 supple-mentation attenuated these increases, particularly in the inactive groups, though this protective effect was abolished by GSK690693 administration. The anabolic markers - serum albumin (an indicator of nutritional status), creatinine (CREA, reflecting muscle mass), and testosterone (crucial for muscle maintenance) - all showed significant decreases in both inactive and protein-restricted conditions, with Rg1 supplementation partially restoring their levels. However, GSK690693 co-administration prevented these Rg1-mediated improvements, suggesting the involvement of AKT pathway in Rg1's protective effects. These findings demonstrate Rg1's capacity to attenuate muscle deterioration and maintain physiological homeostasis under conditions of inactivity and protein restriction, with these benefits being mediated through AKT signaling.

### 3.6 Rg1 Supplementation Preserves Muscle Mass by Modulating Atrophy Signaling and Inflammation

**Fig. (6):**
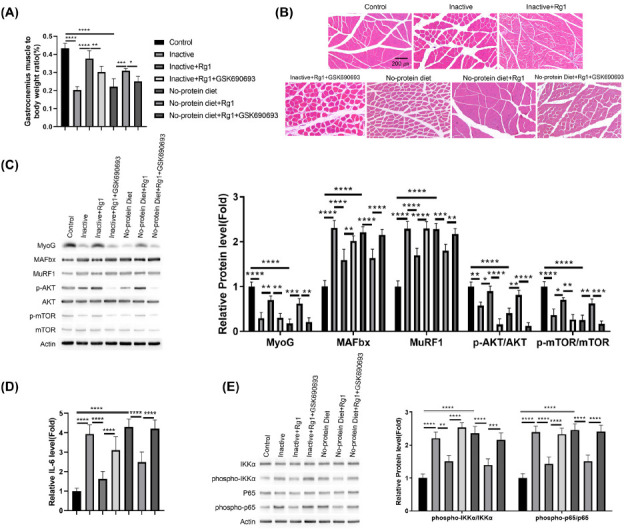
Rg1 Preserves Muscle Mass Through Modulation of Atrophy Signaling and Inflammatory Pathways. Rats were divided into seven groups: Control, Inactive, Inactive+Rg1, Inactive+Rg1+GSK690693, Protein-restricted, Protein-restricted+Rg1, and Protein-restricted+Rg1+GSK690693. (**A**) Ratio of gastrocnemius muscle to body weight. (**B**) Histological examination of muscle fiber dimensions with H&E staining. (**C**) Western blot analysis of MyoG, MAFbx, MuRF1, phosphorylated and total AKT, and mTOR protein levels. (**D**) ELISA analysis of IL-6 levels in muscle tissue. (**E**) Western blot analysis of phosphorylated and total IKKα and p65 protein levels. Actin served as loading control. Data represent mean ± SEM. N=6 animals per group. *p<0.05, **p<0.01, ***p<0.001, **p<0.0001.

To explore the molecular basis of Rg1 supplementation on muscle homeostasis during periods of inactivity and protein restriction, we examined various muscle-related metrics across our experimental groups (Fig. **6**). The gastrocnemius muscle-to-body weight ratio decreased in all experimental conditions, with protein-restricted groups showing the most severe decline. Rg1 supplementation partially reversed this reduction, though this protective effect was abolished by GSK690693 (Fig. **6A**). Histological examination revealed reduced muscle fiber dimensions and increased interstitial space in both inactive and protein-restricted conditions, with protein restriction showing more severe structural disruption. Rg1 supplementation helped maintain muscle fiber structure and size in both conditions, though this protective effect was eliminated by GSK690693 (Fig. **6B**). Western blot analysis demonstrated reduced MyoG levels and elevated atrophy markers (MAFbx and MuRF1) in experimental groups. The phosphorylation of AKT and mTOR was similarly diminished. Rg1 supplementation partially normalized these changes, but its effects were negated by GSK690693 (Fig. **6C**).

We also measured inflammatory markers to assess the anti-inflammatory potential of Rg1. IL-6, a key inflammatory mediator, showed elevated levels in both inactive and protein-restricted conditions. Rg1 supplementation significantly reduced these elevated IL-6 levels, though this effect was prevented by GSK690693 (Fig. **6D**). Further analysis of inflammatory signaling revealed increased phosphorylation of both IKKα and p65 components of the NF-κB pathway in both conditions, despite stable total protein levels. Rg1 treatment effectively suppressed this inflammatory signaling activation, but this suppression was abolished by GSK690693 (Fig. **6E**). These findings suggest that Rg1 preserves muscle mass and reduces atrophy through multiple mechanisms, including maintenance of protein synthesis pathways, suppression of atrophy signaling, and attenuation of inflammatory responses, with these effects being primarily mediated through AKT signaling.

## 4 DISCUSSION

Our findings demonstrate that ginsenoside Rg1 protects against muscle atrophy across multiple pathological conditions, including hyperglycemia, inactivity, and protein restriction. *In vitro*, Rg1 enhanced myoblast survival and protein synthesis under hyperglycemic conditions through AKT/mTOR pathway activation, increasing Myogenin expression while suppressing atrophy-related factors MAFbx and MuRF1. *In vivo* studies confirmed Rg1's protective effects on muscle mass and function, while also revealing its anti-inflammatory properties through suppression of NF-κB signaling. The consistent reversal of these benefits by AKT inhibition establishes this pathway as critical to Rg1's action. These findings position Rg1 as a promising therapeutic agent for muscle atrophy, with potential applications in diabetes management, prolonged immobility, and malnutrition-related conditions.

Ginsenoside Rg1, a primary bioactive compound in ginseng, exhibits diverse therapeutic effects through multiple signaling pathways. Previous studies have demonstrated Rg1's capacity to enhance glucose uptake and insulin sensitivity in muscle cells through AMPK pathway activation [21,26]. In diabetic models, Rg1 shows anti-inflammatory properties and enhances autophagy in pancreatic and splenic tissues [27], while also improving insulin sensitivity through FOXO1 pathway inhibition [28]. Our current findings expand understanding of Rg1's therapeutic potential by demonstrating its protective effects against muscle atrophy under hyperglycemic, inactive, and protein-restricted conditions. This protection is primarily mediated through AKT/mTOR pathway activation, a master regulator of muscle protein homeostasis [29]. The observed upregulation of AKT and mTOR phosphorylation aligns with previous studies of ginseng extracts across various tissues [30]. The dual action of Rg1 on protein homeostasis has been previously documented, with studies showing its ability to both inhibit protein breakdown via PI3K/AKT/FoxO signaling and stimulate protein synthesis through AKT/mTOR activation in C2C12 cells under starvation conditions [31,32]. Our research provides novel evidence that this same mechanism operates under hyperglycemic conditions, preserving muscle cell viability and protein synthesis through AKT/mTOR pathway modulation.

A key finding of our study is Rg1's capacity to enhance Myogenin expression while suppressing muscle atrophy-related E3 ligases (MAFbx and MuRF1) under hyperglycemic conditions. This extends previous observations of Rg1's protective effects against starvation-induced protein degradation [31] to a hyperglycemic context, particularly relevant for diabetes where muscle progenitor cell function and regeneration are typically impaired [33]. The molecular mechanism underlying these effects involves the PI3K/Akt/mTOR signaling axis [34]. Activation of this pathway promotes Myogenin expression while simulta-neously suppressing MAFbx and MuRF1 transcription through FoxO transcription factor phosphorylation and nuclear exclusion [35]. Our demonstration that Rg1 activates the AKT/mTOR pathway provides a mechanistic explanation for its dual effect on myogenic and atrophy-related factors. These findings highlight the potential of Rg1 as an adjunct therapy for diabetic muscle atrophy, addressing a significant yet often overlooked complication of diabetes.

Furthermore, the efficacy of Rg1 in attenuating muscle loss in both inactivity and protein restriction models underscores its versatility as a potential therapeutic agent. This broad efficacy parallels other natural compounds like resveratrol and epigallocatechin gallate, which have shown promise in treating disuse atrophy [36,37]. The anti-inflammatory properties of Rg1, evidenced by reduced IL-6 levels and suppressed NF-κB signaling, coupled with decreased atrophy markers and preserved muscle function, suggest significant therapeutic potential. These effects are particularly relevant for clinical scenarios involving prolonged bed rest or malnutrition, where current therapeutic options remain limited [38].

## STUDY LIMITATIONS

Several limitations of our study warrant further investigation. First, while we demonstrated Rg1's protective effects against hyperglycemia-induced muscle atrophy *in vitro*, validation in diabetic animal models is necessary to fully establish its therapeutic potential in diabetes-related muscle complications. Second, although we identified AKT/mTOR pathway activation as crucial for Rg1's effects, the upstream mechanisms by which Rg1 initiates this signaling cascade remain unclear and require further elucidation. Third, our study focused primarily on undifferentiated myoblast cells, yet satellite cells play essential roles in muscle homeostasis and repair. Future studies should examine Rg1's effects on satellite cell function, acti-vation, and muscle fiber regeneration under patholo-gical conditions. These investigations would provide a more comprehensive understanding of Rg1's thera-peutic potential and guide its clinical application.

## CONCLUSION

To sum up, our study demonstrates the protective effects of ginsenoside Rg1 against muscle atrophy in hyperglycemic conditions, inactivity, and protein restriction. Rg1 mitigates muscle wasting by activating the AKT/mTOR pathway, enhancing Myogenin expression, and suppressing atrophy-related genes MAFbx and MuRF1, as observed in both C2C12 myoblasts and rat models. These findings highlight Rg1's potential as a therapeutic agent for various muscle-wasting conditions. While further research is needed to fully elucidate its mechanisms and clinical efficacy, this study provides a strong foundation for developing Rg1-based interventions to combat muscle atrophy in diverse pathological scenarios.

## Data Availability

The data generated in this study are available upon request to the corresponding author.

## LIST OF ABBREVIATIONS

MyoG: Myogenin
SD: Standard Deviation
AST: Aspartate Aminotransferase
ALT: Alanine Aminotransferase
